# Supplementary material for: Analysis of In Vivo Transcriptome of Intracellular Bacterial Pathogen Salmonella enterica serovar Typhmurium Isolated from Mouse Spleen
Source: Pathogens. 2021 Jun 30;10(7):823. doi: 10.3390/pathogens10070823 (PMC8308634; doi:10.3390/pathogens10070823)
Supplement: Supplementary file 1 [file pathogens-10-00823-s001.zip › pathogens-1273672 supp figures.pdf]

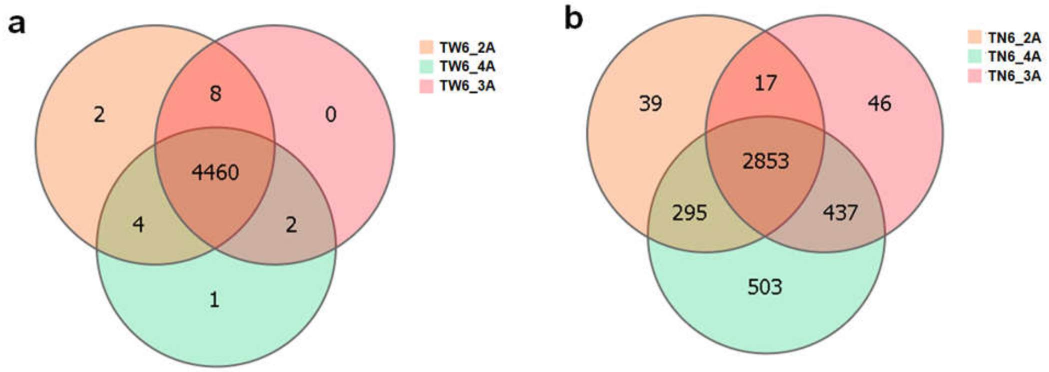


**Supplementary Figure S1.** Venn diagrams showing gene expression profiles. Venn diagrams were created to quantify the overlap between the three biological replicates. (**a**) 4477 transcripts were detected the *in vitro* group, and 4460 genes were identified among the three biological replicates. (**b**) 3737 transcripts were detected in the *in vivo* group, of which 2853 genes were identified among the three biological replicates.

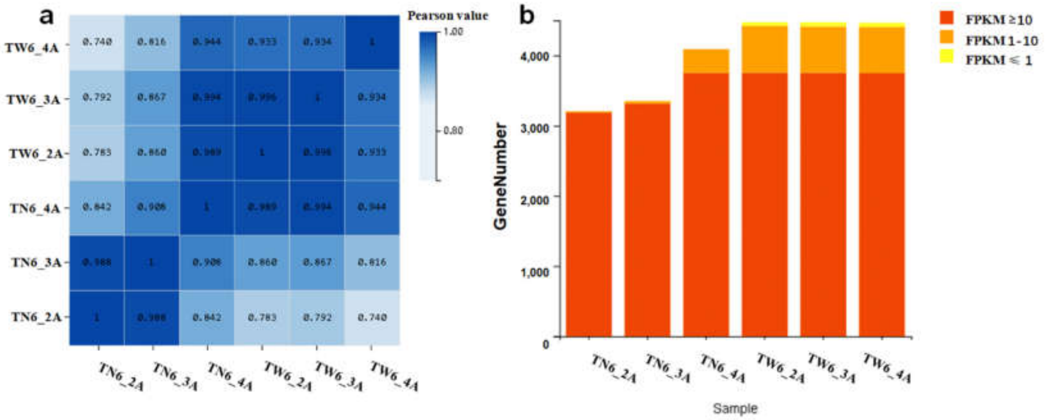


**Supplementary Figure S2.** Correlation heatmap and expression quantity accumulation diagram. (**a**) The X and Y axes represent each sample. The color represents the correlation coefficient (the darker the color, the higher the correlation; the lighter the color, the lower the correlation). The correlation coefficient between the *in vivo* samples was more than 84.2%, and between the *in vitro* samples was more than 93.3%. (**b**) X axis represents the name of the sample, Y axis represents the number of genes. The depth of color represents different expression levels: genes with a very low expression level of FPKM ≤ 1, genes with a low expression level of FPKM between 1 and 10, and genes with a medium or high expression level of FPKM ≥ 10.
